# Prognostic value of MRI in arthroscopic treatment of chronic patellar tendinopathy: a prospective cohort study

**DOI:** 10.1186/s12891-017-1508-2

**Published:** 2017-04-04

**Authors:** Peter Ogon, Kaywan Izadpanah, Helge Eberbach, Gernot Lang, Norbert P. Südkamp, Dirk Maier

**Affiliations:** 1 Center of Orthopedic Sports Medicine, Breisacher Strasse 84, 79110 Freiburg, Germany; 2 Department of Orthopedics and Trauma Surgery, Medical Center - Albert-Ludwigs-University of Freiburg, Faculty of Medicine, Albert-Ludwigs-University of Freiburg, Hugstetter Strasse 55, 79106 Freiburg, Germany

**Keywords:** Patellar tendinitis, Jumper’s knee, MRI, Prognosis, Outcome, Return to sports

## Abstract

**Background:**

To date, prognostic outcome factors for patients undergoing arthroscopic treatment due to chronic patellar tendinopathy (PT) are lacking. The purpose of this study was to investigate whether preoperatively assessed MRI parameters might be of prognostic value for prediction of functional outcome and return to sports in arthroscopic treatment of chronic PT.

**Methods:**

A prospective cohort study was conducted including 30 cases (4 female and 24 male competitive athletes) undergoing arthroscopic patellar release (APR) due to chronic PT. The mean age was 28.2 years (range, 18–49 years) at the time of surgery, and the mean follow-up period was 4.2 years (range, 2.2–10.4 years). Preoperatively assessed MRI parameters included bone marrow edema (BME) of the inferior patellar pole, patellar tendon thickening, infrapatellar fat pad (IFP) edema, and infrapatellar bursitis. Prevalences of preoperative MRI findings were correlated to functional outcome scores in order to determine statistically significant predictors.

**Results:**

All athletes regained their preinjury sports levels. Athletes featuring preoperative IFP edema showed significantly inferior modified Blazina score (0.6 ± 0.7 vs. 0.2 ± 0.5), single assessment numeric evaluation (SANE; 86.0 ± 8.8 vs. 94.3 ± 7.5), and Visual Analogue Scale (VAS; 1.0 ± 1.2 vs. 0.3 ± 0.8) compared to subjects without IFP edema (*p* < 0.05). Return to sports required a mean of 4 ± 3.2 months. On average, patients with IFP edema needed significantly more time to return to sports than subjects without IFP edema (6.5 vs 2.8 months; *p* < 0.05). The simultaneous presence of BME and IFP edema was associated with significantly inferior outcomes by means of the Victorian Institute of Sport Assessment questionnaire for patients with patellar tendinopathy (VISA-P; 88.1 ± 11.9 vs. 98.6 ± 4.2), SANE (84.3 ± 10.2 vs. 93.1 ± 8.3), and VAS (1.3 ± 1.4 vs. 0.3 ± 0.9) compared to an isolated BME or isolated IFP edema.

**Conclusions:**

This is the first study identifying prognostic outcome factors in arthroscopic treatment of chronic PT. Preoperative IFP edema alone or simultaneous BME and IFP edema on preoperative MRI were associated with inferior functional outcome and delayed return to sports. Knowledge of these predictive factors might improve risk stratification, individualize treatment and postoperative rehabilitation, and contribute to improve clinical outcome. Moreover, current findings offer the potential for novel therapeutic approaches.

## Background

Anterior knee pain and tenderness of the proximal patellar tendon insertion site are frequently described characteristics of patellar tendinopathy (PT) [4, 29]. Given its high prevalence in jumping sports, Blazina et al. [4] introduced the term of “jumper’s knee” as a synonym for PT. The overall prevalence of PT yields 14% in national elite athletes and increases up to 30 to 45% in high-risk sports such as basketball and volleyball [10, 17, 19]. Reduced muscular flexibility has been determined as an intrinsic risk factor for PT in athletes [2, 41]. Additionally, PT is commonly observed in older and obese patients as well as in subjects exhibiting an increased vastus medialis muscle size [9, 33, 34]. Some investigators believe that the inferior pole of the patella has a propensity to impinge upon the deep surface of the proximal patellar tendon during knee flexion [14, 21, 33]. It is assumed, that a longer non-articular inferior patellar pole might be a risk factor for the onset of PT. On the contrary, Schmid and co-workers found identical biometric relationships when comparing tendon-patella-dimensions in symptomatic and asymptomatic individuals and therefore rather consider chronic overload than bony impingement as the origin of pain [32]. Furthermore, Culvenor et al. [7] demonstrated that an increased infrapatellar Hoffa fat pad (IFP) might potentially contribute to the pathogenesis of PT.

Beside patient history and clinical findings, ultrasonography, conventional radiographs, and MRI are routinely used for the diagnosis of PT as well as exclusion of concomitant pathologies [14, 28, 38]. Typical MRI findings in PT are bone marrow edema (BME) of the inferior patellar pole, thickening of the proximal patellar tendon, abnormal signal intensity of the proximal patellar tendon, infrapatellar bursitis, and hypertrophy as well as IFP edema [5, 7, 18, 27, 28, 33]. Both, open and arthroscopic surgical treatment proved to be effective as treatment for chronic-refractory PT unresponsive to non-operative approaches [20]. Nowadays, arthroscopic techniques are favored due to less invasiveness and faster rehabilitation [22, 23, 30].

While the pathogenesis and risk factors of PT are well understood, evidence on factors predictive of postoperative outcome are lacking [37]. We hypothesized, that certain preoperative MRI findings may have prognostic value for postoperative outcome in patients undergoing arthroscopic treatment of chronic PT. Such knowledge would allow for improved estimation of postoperative functional and clinical outcome as well as time required for return to sports. Consequently, therapeutic management guided by prognostic factors could potentially translate into optimized and accelerated treatment and rehabilitation of athletes.

The aim of the present study was to analyze the prognostic value of morphologic MRI parameters for the clinical and functional outcome of competitive athletes undergoing arthroscopic treatment due to chronic PT unresponsive to non-operative therapy [25]. Therefore, we quantified preoperative MRI findings specific for chronic PT and correlated them to functional and clinical outcome measures.

## Methods

### Study population

This study was approved by the Ethics Committee of the University of Freiburg (Vote-Nr.: 584/16). All participating patients provided written informed consent. Between 07/2005 and 03/2011, a prospective, prognostic cohort study was performed on patients undergoing arthroscopic patellar release (APR) due to chronic-refractory, symptomatic PT. Inclusion criteria were: age > 18 years, competitive athlete, operative treatment by means of APR, chronic-refractory, symptomatic PT despite a minimum period of 6 months of non-operative treatment, and a postoperative follow-up period > 2 years. Patients displaying abnormal signal intensity indicating partial rupture of the proximal patellar tendon and concomitant intra- or extra-articular knee joint pathologies (e.g., patellofemoral malalignment/maltracking, chondral lesions >1° according to the International Cartilage Repair Society, meniscal tears and ligamentous injuries) were excluded. Further exclusion criteria were impairment of sports performance and/or sports cessation for reasons other than symptomatic PT (e.g. secondary injuries of the affected extremity), incompliance and use of doping agents. All patients were referred to our institution for operative treatment due to failed conservative treatment. Non-operative measures consisted of eccentric physiotherapy for a minimum period of 3 months, oral non-steroidal anti-inflammatory drugs, extracorporeal shockwave treatment and up to 3 ultrasound-guided peritendinous corticosteroid injections. The first author (PO) performed all surgeries using an identical operative technique and postoperative rehabilitation program as described previously [25].

A total of 29 patients were eligible for study participation, and all of them could be included. After study inclusion, one patient had to be excluded due to an ipsilateral ankle fracture resulting in a permanent restriction of function. Follow-up examinations were completed in 28 patients (96.6%). Two male athletes were operated bilaterally corresponding to a total of 30 cases. Sports involved were running (*n* = 10), soccer (*n* = 8), handball (*n* = 4), alpine skiing (*n* = 3), cycling (*n* = 2), and body building (*n* = 1). None of the athletes experienced an injury of the index knee prior to surgery.

### Preoperative diagnostics

Preoperatively, one experienced orthopedic surgeon obtained medical histories and performed standardized clinical examination of all knee joints. Radiological diagnostics included plain radiographs (anteroposterior and lateral knee views, axial patellofemoral views), ultrasonography and magnetic resonance imaging (MRI) of the affected knee joints to exclude intra- and extraarticular co-pathologies.

### Preoperative MRI evaluation

All MRI examinations were performed according to the guidelines of the German Medical Association and German Roentgen Society. The detailed MRI protocol is displayed in Table 1.

**Table 1:** Preoperative MRI protocol

Pulse sequence	Type	Repetition/Echo time (ms)	Echo-train length	Bandwidth (kHz)	Field of view (mm)	Image matrix (pixels)	Number of excitations	Section thickness (mm)	Spacing (mm)
1	Sagittal proton density-weighted sequence	3000-4070/33,5-40	10	42	200	512 × 512	2	3.0	3.3
2	Axial proton density-weighted sequence	3550/33	10	25	180	1024 × 1024	1	3.0	3.3
3	Coronal proton density-weighted sequence	3000-4070/33,5-40	10	42	180	1024 × 1024	2	3.0	3.3
4	Sagittal T1 weighted sequence	400/14,7	6	21	180	512 × 512	2	3	3.2

Proton density-weighted spin-echo sequences with spectral fat saturation were acquired without administration of contrast agents.

**Fig. 1:**
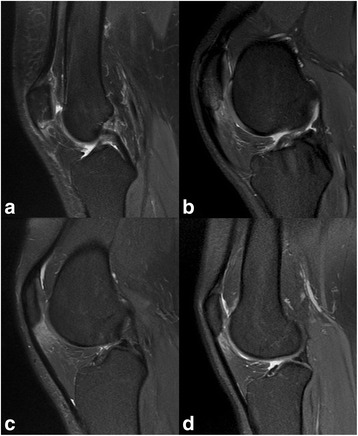
Specific MRI findings in chronic PT: **a** BME of the inferior patellar pole, **b** thickening of proximal patellar tendon, **c**/**d** IFP edema with infrapatellar bursitis

Preoperative MRI evaluation of the patellar tendon and surrounding tissues were conducted utilizing T1, T2, and proton density-weighted sequences [24–26]. Echo times were ranging between 33 ms and 40 ms to avoid “magic-angle” artifacts [15]. Knee MRIs were assessed by an independent senior radiologist having profound knowledge of MRI changes associated with PT. Standardized MRI evaluation included assessment of the following criteria: 1.) BME of the inferior patellar pole (Fig. 1a), 2.) IFP edema (Fig. 1c/d), 3.) infrapatellar bursitis (Fig. 1c/d) and 4.) thickening of the proximal patellar tendon (Fig. 1b).

BME of the inferior patellar pole was defined as distinct, focal enhancement compared to a normal surrounding bone signal. Accordingly, IFP edema was identified, if the signal intensity of the IFP revealed a clearly detectable increase compared to normal fat tissue. Infrapatellar bursitis was defined as pathological fluid collection within the infrapatellar bursa. Thickening of the patellar tendon was noted, if the patellar tendon’s proximal portion displayed a non-harmonic swelling compared to the distal part of the tendon, exceeding an AP-diameter > 7 mm [5, 18, 27, 28, 38].

### Outcome measures

Standardized follow-up examinations were performed by an independent senior orthopaedic resident. The validated German version of the VISA-P score (0–100 points) and the modified Blazina score according to Ferretti et al. [11] served as functional outcome scores [20]. The modified Blazina score represents a pathology-specific outcome measure comprising five stages according to symptoms occurring at different level of sports/activity (0 = no pain, 1 = pain after intense sports activity, 2 = pain at beginning of and after sports activity, 3 = pain during activity at a satisfactory level, 4 = pain during sports activity at an non-satisfactory level, and 5 = pain during daily activity. Athletes rated the subjective function of the affected knee by means of single assessment numeric evaluation (SANE), as determined by the written response to the following question: On a scale from zero to 100, how would you rate your knee today (100 being normal) [40]?

Additionally, patients were advised to document their maximum pain level during activity on a visual analog scale (VAS). Postoperatively, the number of months was quantified until athletes were able to perform specific exercises without any or minimal pain as defined by Kelly et al. [16]. At follow-up, each athlete was asked to assess his symptoms and level of sports performance compared to before injury.

### Statistics

Statistical analysis was performed using the software SAS, version 9.3 (SAS Institute, Cary, North Carolina, USA). Descriptive results are given as mean values with standard deviations (±) and/or ranges. A non-parametric Wilcoxon test was used to compare subgroups of patients with negative and positive MRI findings, respectively. Categorical parameters were analyzed with the exact Fisher test. Statistical significance was considered for *p*-values < 0.05.

## Results

### Clinical outcome

There were no perioperative or surgical complications. One senior orthopedic surgeon performed clinical examination on the first postoperative day prior to discharge. All athletes stated relief of their previous sharp and circumscribed pain around the inferior patellar pole. They were already able to perform a one-legged stance and some light squats without experiencing their previous characteristic pain of PT.

#### Outcome scores

At follow-up, all outcome measures showed significant improvements compared to their preoperative values.

The VISA-P score increased from a preoperative mean value of 55.6 points (±12.4 points) to a mean follow-up value of 95.4 points (±8.1 points; *p* < 0.0001). Twenty-one (70.0%) cases achieved the full VISA-P score of 100 points. Overall, 22 (73.3%) cases achieved excellent (91 to 100 points), 7 (23.3%) cases good (81 to 90 points) results, and one (3.3%) patient an unsatisfactory (70 points) outcome according to the VISA-P score.

The mean modified Blazina score decreased from 4.1 points (±0.77 points) to 0.3 points (±0.60 points; *p* < 0.0001). Twenty-three (76.7%) cases experienced no pain at all (0 points), 5 (16.7%) felt minor to moderate pain after intense sports activity (1 point) and 2 (6.7%) had pain at the beginning of and after sports activity (2 points).

Likewise, patients experienced a significant improvement of their subjective knee function indicated by an increase of SANE score from 45.2 ± 17.5 preoperatively to 90.2 ± 11.4 at last follow-up (*p* < 0.0001). Fourteen (46.7%) patients rated their subjective knee function as excellent (91 to 100), 12 (40.0%) as good (81 to 90), 2 (6.7%) as satisfactory (71 to 80), and 2 (6.7%) as still unsatisfactory with a score of 70 in both cases. Still, both patients were able to perform sports at their preinjury competition level.

Moreover, the mean preoperative pain level according to VAS decreased from 5.7 (±1.30) to 0.5 (±1.01; *p* < 0.0001) at follow-up.

#### Return to sports

The mean time period required for return to sports was 4.0 months (±3.2 months; range, 0.5- 12 months). At follow-up, all athletes were able to perform at their preinjury, competitive sports level. Twenty-three (76.7%) cases did not experience any symptoms during or after sports activity. Five (16.7%) athletes felt minor to moderate pain after intense sports performance. The two (6.7%) athletes with an unsatisfactory subjective result complained about notable pain at the onset of and/or after sports competition. However, both were able to compete in sports at their previous level and local symptoms were less distinct.

### MRI findings

Pathological thickening of the proximal patellar tendon (Fig. 1b) was noted in all cases. Four cases displayed infrapatellar bursitis with pathological fluid collection (Fig. 1c/d). Table 2 summarizes sports, age and MRI findings specific for chronic PT for all 30 cases.

**Table 2:** Basic demographics and case-related MRI findings

Case	Sports	Age	BME	Tendon Thickening	IFP Edema	Infrapatellar Bursitis
1	Running	30	yes	yes	yes	no
2	Soccer	23	yes	yes	yes	no
3	Handball	29	no	yes	no	no
4	Handball	28	no	yes	no	no
5	Alpine skiing	19	yes	yes	no	no
6	Soccer	22	yes	yes	no	no
7	Cycling	33	yes	yes	no	no
8	Handball	19	no	yes	no	no
9	Soccer	30	no	yes	no	no
10	Running	34	yes	yes	yes	no
11	Running	49	no	yes	yes	no
12	Running	39	yes	yes	no	no
13	Hammer Throw	24	yes	yes	no	no
14	Running	43	yes	yes	yes	yes
15	Soccer	30	no	yes	yes	yes
16	Body Building	43	no	yes	no	no
17	Soccer	23	yes	yes	yes	no
18	Handball	19	yes	yes	no	no
19	Soccer	20	no	yes	no	no
20	Running	31	yes	yes	no	yes
21	Running	29	no	yes	no	no
22	Running	41	no	yes	no	yes
23	Alpine Skiing	20	yes	yes	no	no
24	Running	21	no	yes	no	no
25	Cycling	39	yes	yes	yes	no
26	Soccer	21	yes	yes	yes	no
27	Soccer	27	yes	yes	no	no
28	Alpine Skiing	24	yes	yes	no	no
29	Running	34	no	yes	no	no
30	Running	22	no	yes	yes	no
Total cases with positive MRI	17			30	10	4

#### Bone marrow edema

Preoperatively, BME of the inferior patellar pole was present in 17/30 (56.7%) cases and absent in 13/30 (43.3%) cases. Patients presenting a BME were not associated with impaired clinical or functional outcome at the last follow-up (*p* > 0.05). Table 3 shows specific profiles and outcomes for both groups.

**Table 3:** Outcome analysis related to presence of BME

	BME present	BME absent	*P*-Value
Case Number [n, (%), total *n* = 30]	17 (56.7%)	13 (43.3%)	
Mean Age at Surgery [years]	27.4 (±8.0)	29.3 (±8.5)	*P* = 0.570
Mean Follow-up [years]	3.2 (±1.3)	5.3 (±3.8)	*P* = 0.107
VISA-P score preop. [points]	53.9 (±12.5)	58.8 (±12.7)	*P* = 0.308
VISA-P score postop. [points]	94.1 (±9.6)	97.2 (±5.3)	*P* = 0.327
Mod. Blazina score preop. [points]	4.0 (±0.9)	4.2 (±0.6)	*P* = 0.672
Mod. Blazina score postop. [points]	0.4 (±0.7)	0.2 (±0.4)	*P* = 0.335
Subjective Knee Function (SANE) preop.	44.7 (±18.2)	45.4 (±18.0)	*P* = 0.833
Subjective Knee Function (SANE) postop.	90.0 (±10.6)	93.5 (±5.2)	*P* = 0.617
Pain (VAS) preop.	5.7 (±1.4)	5.8 (±1.2)	*P* = 0.949
Pain (VAS) postop.	0.7 (±1.2)	0.2 (±0.6)	*P* = 0.226
Mean Time Period for Return to Sports [months]	4.1 (±3.2)	3.9 (±3.3)	*P* = 0.780

#### Infrapatellar fat pad edema

IFP edema was detected in 10/30 (33.3%) of cases on preoperative MRI. Patients with IFP edema had a significantly lower mean VISA-P score and subjective knee function (SANE) preoperatively compared to unaffected subjects. At the last follow-up, presence of IFP edema was associated with significantly inferior clinical outcome by means of modified Blazina score, SANE and VAS and also longer return to sports periods (6.5 vs. 2.8 months) compared to patients without IFP edema. Table 4 shows group-specific profiles and outcomes of patients with and without IFP edema.

**Table 4:** Outcome analysis related to presence of IFP edema

	IFP edema present	IFP edema absent	*P*-Value
Case Number [n, (%), total *n* = 30]	10 (33.3%)	20 (66.7%)	
Mean Age at Surgery [years]	30.9 (±10.4)	26.9 (±6.6)	*P* = 0.270
Mean Follow-up [years]	3.5 (±1.5)	4.5(±3.4)	*P* = 0,555
VISA-P score preop. [points]	**48.3 (±9.4)**	**59.9(±12.3)**	***P*** = 0.024*
VISA-P score postop. [points]	91.7 (±11.3)	97.3 (±5.3)	*P* = 0.184
Mod. Blazina score preop. [points]	4.4 (±0.7)	3.9 (±0.8)	*P* = 0.106
Mod. Blazina score postop. [points]	**0.6 (±0.7)**	**0.2 (±0.5)**	***P*** = 0.024*
Subjective Knee Function (SANE) preop.	**33.5 (±16.0)**	**50.8 (±16.0)**	***P*** = 0.016*
Subjective Knee Function (SANE) postop.	**86.0 (±8.8)**	**94.3 (±7.5)**	***P*** = 0.009*
Pain (VAS) preop.	6.5 (±1.5)	5.4 (±1.0)	*P* = 0.054
Pain (VAS) postop.	**1.0 (±1.2)**	**0.3 (±0.8)**	***P*** = 0.009*
Mean Time Period for Return to Sports [months]	**6.5 (±3.8)**	**2.8 (±2.0)**	***P*** = 0.003*

*: indicates statistical significance with P< 0.05

#### Simultaneous bone marrow edema and infrapatellar fat pad edema

A proportion of 7/30 (23.3%) cases simultaneously showed both BME and IFP edema, whereas 13/30 (43.3%) cases only displayed an isolated edema (BME or IFP edema). Patients having such combined edemas had a significantly lower mean preoperative VISA-P score compared to patients with an isolated edema. At follow-up, simultaneous presence of BME and IFP was associated with significantly inferior functional and clinical outcome by means of VISA-P, SANE and VAS score compared to subjects with an isolated edema. Though, the mean time required for return to sports did not differ significantly. Table 5 shows group-specific profiles and outcomes of cases with combined edemas compared to cases with an isolated edema.

**Table 5:** Outcome analysis related to simultaneous presence of BME and IFP edema

	BME and IFP edema present	BME or IFP edema present	*P*-Value
Case Number [n, (%), total *n* = 30]	7 (23.3%)	13 (43.3%)	
Mean Age at Surgery [years]	29.7 (±9.7)	27.6 (±8.8)	*P* = 0.578
Mean Follow-up [years]	3.3 (±1.4)	3.3 (±1.5)	*P* = 0.888
VISA-P score preop. [points]	**46.0 (±7.8)**	**58.1(±12.1)**	***P*** = 0.043*
VISA-P score postop. [points]	**88.1 (±11.9)**	**98.6 (±4.2)**	***P*** = 0.028*
Mod. Blazina score preop. [points]	4.4 (±0.8)	3.8 (±0.9)	*P* = 0.176
Mod. Blazina score postop. [points]	0.7 (±0.8)	0.2 (±0.6)	*P* = 0.085
Subjective Knee Function (SANE) preop.	35.0 (±15.5)	46.5 (±19.5)	*P* = 0.197
Subjective Knee Function (SANE) postop.	**84.3 (±10.2)**	**93.1 (±8.3)**	***P*** = 0.049*
Pain (VAS) preop.	6.4 (±1.4)	5.5 (±1.5)	*P* = 0.181
Pain (VAS) postop.	**1.3 (±1.4)**	**0.3 (±0.9)**	***P*** = 0.022*
Mean Time Period for Return to Sports [months]	5.9 (±3.2)	4.1 (±3.9)	*P* = 0.071

*: indicates statistical significance with P< 0.05

## Discussion

This prospective cohort study identified specific preoperative MRI findings serving as prognostic outcome factors in arthroscopic treatment of chronic PT. Preoperative IFP edema was associated with significantly inferior outcomes related to the modified Blazina score, subjective knee function (SANE) and VAS. All athletes returned to their preinjury, competitive sports levels after a mean of 4.0 months (±3.2 months). Return to sports was significantly delayed in athletes with preoperative IFP edema (6.5 versus 2.8 months). Moreover, coexistence of BME and IFP edema showed significantly inferior outcomes related to the VISA-P score, SANE and VAS compared to singular edema (BME or IFP), but did not prolong time required for return to sports.

Multiple studies focused on risk factors of PT [9, 34, 37, 41]. However, no previous study identified evidence-based prognostic outcome factors. Current study found preoperative IFP edema to be a determinant prognostic factor being associated with inferior functional outcomes and prolongation of return to sports. This finding supports the current understanding towards the contribution of peritendinous soft tissue, such as tenosynovium and IFP to the pathogenesis of chronic PT [7, 12, 13, 26, 31, 36]. With respect to the patellar tendon, the IFP effects stress distribution at the enthesis and provides relevant neurovascular supply [3, 12, 26]. Moreover, the IFP is known to trigger complex pathophysiologic and biochemical processes in response to osteoarthritis and other acute and chronic disorders of the knee joint [13, 36]. PT involves a complex interaction of extrinsic and intrinsic processes. Chronic mechanical overload is supposed to cause microinjuries of the patellar tendon enthesis which ultimately leads to an inflammatory reaction of the surrounding soft tissues promoting neovascularization, nerve ingrowth into the proximal patellar tendon, and increased pain sensation [1, 7, 8, 18, 31, 35, 36]. In the present study, this pathologic cycle of metabolic and proinflammatory alterations was represented by IFP edema and BME assessed by MRI examinations and resulted in inferior pre- and postoperative function, higher pain levels and longer periods of postoperative recovery [13, 36]. Furthermore, our findings confirm MRI observations by Culvenor and Warden et al. [7, 38] who detected associations between MRI-based IFP abnormalities and clinical symptoms and proposed that a hypertrophic IFP would promote the perception and ﻿recalcitrance﻿ of pain. In the study of Warden et al. [38] a proportion of 13/30 (43.3%) symptomatic patients showed IFP signal alterations indicating an edematous reaction. These findings correspond well to ours with 10/30 (33.3%) cases presenting an IFP edema.

The procedure of APR focuses on causative treatment of peritendinous soft tissue pathologies (IFP, tenosynovitis) without performing any tendon or bone resection [25]. Present study shows, that APR as a minimal-invasive soft tissue procedure yields favorable results which are equivalent to more invasive (arthroscopic or open) procedures involving tendon and/or bone resection [6, 22, 24]. These findings are confirmed by the study of Willberg et al. [39], who performed arthroscopic shaving in order to address neovascularization and neoinnervation adjacent to the tendinopathic changes on the posterior aspect of the tendon. Spur formation and/or increased extension of the inferior patellar pole with secondary bony impingement used to be regarded as a predominant pathogenetic factor in PT. Consequently, many authors proposed inferior patellar pole resection as a causative therapy. Though, current research raises increasing doubts towards this hypothesis [7, 32].

In clinical practice, knowledge of predictive MRI findings could prove valuable for a more detailed risk stratification and prognosis of outcome especially in patients with high functional demands such as competitive athletes. Additionally, prognostic factors can serve for the purpose of individualized treatment and rehabilitation concepts potentially resulting in improved clinical outcome. In the present study, athletes needed 4 months on average to return to competitive sports levels. We assume that chronic muscular and kinematic disorders may have additionally contributed to the prolongation of postoperative rehabilitation. In our experience, athletes should refrain from sports for at least 3 months after APR. A too progressive rehabilitation might promote (partial) persistence, prolongation, and/or recurrence of symptoms. Results of current research enable the potential for novel therapeutic approaches. Specific conservative treatment for IFP edema might accelerate postoperative rehabilitation and improve clinical outcome following arthroscopic treatment of PT.

A few limitations need to be considered. We ruled out patients with signal alterations of the proximal patellar tendon since partial tendon rupture could not be reliably excluded. Thus, present findings only apply to patients with structurally intact patellar tendons. Additionally, the present study design did not appreciate the potential influence of IFP volume within a sub-analysis as demonstrated previously [7]. Postoperative MRIs were not performed routinely, and thus could not be analyzed for follow-up assessment. Furthermore, the relatively small study population did not allow a sports-specific sub-analysis. These issues need to be addressed in future studies analyzing the causes of IFP edema (i.e. type of sport, level of competition, modality of conservative treatment, comorbidity, and duration of symptoms). However, to our best knowledge, there exists no previous study focusing on prognostic outcome factors in arthroscopic treatment of PT.

## Conclusions

This is the first study identifying prognostic outcome factors in arthroscopic treatment of chronic PT. Preoperative IFP edema alone or IFP edema and concomitant BME on preoperative MRI were associated with inferior functional outcome and delayed return to sports. Current findings emphasize the pathogenetic importance of peritendinous soft tissue involvement in chronic symptomatic PT being effectively addressed by the minimal-invasive technique of APR without performing bone or tendon resection. Knowledge of these predictive factors might improve risk stratification, individualize treatment and postoperative rehabilitation, and contribute to improve clinical outcome. Moreover, current findings offer the potential for novel therapeutic approaches.

## Abbreviations

APR: Arthroscopic patellar release
BME: Bone marrow edema
IFP: Infrapatellar fat pad
MRI: Magnetic resonance imaging
PT: Patellar tendinopathy
SANE: Singular assessment numeric evaluation
VAS: Visual analog scale
